# Identification and validation of potential shared diagnostic markers for sepsis-induced ARDS and cardiomyopathy via WGCNA and machine learning

**DOI:** 10.3389/fmolb.2025.1665387

**Published:** 2025-11-11

**Authors:** Jing Wei, Baoyue Huang, Kunlin Hu, Bin Xiong, Shulin Xiang

**Affiliations:** 1 Department of Intensive Care Unit, The People’s Hospital of Guangxi Zhuang Autonomous Region, Nanning, Guangxi Zhuang Autonomous Region, China; 2 Research Center of Communicable and Severe Diseases, The People’s Hospital of Guangxi Zhuang Autonomous Region, Nanning, Guangxi Zhuang Autonomous Region, China; 3 Guangxi Clinical Research Center Construction Project for Critical Treatment of Major Communicable Diseases, Nanning, Guangxi Zhuang Autonomous Region, China

**Keywords:** sepsis-induced ALI/ARDS, septic cardiomyopathy, SOCS3, immune infiltration, molecular docking

## Abstract

**Background:**

Sepsis frequently results in complications such as acute respiratory distress syndrome (ARDS) and cardiomyopathy. This study aims to identify common diagnostic markers and elucidate the underlying mechanisms of these sepsis-induced complications.

**Methods:**

We obtained datasets related to ARDS and sepsis-induced cardiomyopathy (SIC) from the GEO database and applied weighted gene co-expression network analysis (WGCNA) to identify differentially expressed genes (DEGs), which were integrated with key module genes. Feature genes were selected using support vector machine-recursive feature elimination (SVM-RFE) and random forest (RF) algorithms. An artificial neural network (ANN) model was constructed and its diagnostic performance was evaluated using receiver operating characteristic (ROC) curves. Machine learning algorithms effectively identified key hub genes associated with sepsis-induced ARDS and cardiomyopathy, with their robustness validated through ROC analysis. A cellular model of sepsis-induced lung injury was employed to examine hub gene expression. Additionally, we investigated inflammation and immune responses by characterizing immune landscapes using CIBERSORT and performing correlation analyses among feature genes, immune infiltration, and clinical characteristics. Finally, potential small-molecule compounds were identified from the PubChem database.

**Results:**

Five key genes—LCN2, AIF1L, STAT3, SOCS3 and SDHD—were identified. SOCS3 showed strong diagnostic potential with gene set enrichment analysis (GSEA) highlighting its role in biological processes and immune responses. SOCS3 expression correlated strongly with immune cells. Dexamethasone, resveratrol and curcumin were identified as potential SOCS3-targeting drugs.

**Conclusion:**

Five genes were identified as diagnostic biomarkers for sepsis-induced ARDS and cardiomyopathy, with SOCS3 serving as a key hub gene and potential therapeutic target.

## Introduction

Sepsis, a systemic and dysregulated response to infection, represents a major global health challenge due to its high prevalence and mortality rates. This complex syndrome triggers a cascade of pathophysiological responses, leading to multiple organ dysfunction. Among its severe complications, sepsis-induced acute respiratory distress syndrome (ARDS) and sepsis-induced cardiomyopathy (SIC) are particularly common and devastating (Singer et al., 2016; Rudd et al., 2020).

Acute respiratory distress syndrome (ARDS) results from severe pulmonary inflammation and extensive alveolar damage. It is characterized by widespread infiltration of inflammatory cells and increased permeability of the alveolar–capillary barrier, leading to pulmonary edema and hypoxemia (Gorman et al., 2022). Sepsis-induced cardiomyopathy (SIC) currently lacks a uniform definition but is generally described as an acute syndrome of nonischemic cardiac dysfunction associated with sepsis. Factors such as myocardial suppression, mitochondrial dysfunction, calcium homeostasis imbalance and cytokine overload collectively impair cardiac function (Martin et al., 2019). These complications in the heart and lungs increase mortality risk and complicate management, necessitating advanced clinical interventions. Despite extensive research, the molecular mechanisms involved remain incompletely understood, highlighting the urgent need for innovative diagnostic and therapeutic strategies. Therefore, identifying diagnostic biomarkers for sepsis-related vital organ dysfunction and developing targeted therapeutic approaches are crucial for improving patient survival rates (Coopersmith et al., 2018).

While biomarkers such as C-reactive protein and procalcitonin are widely used for diagnosing and evaluating sepsis (Yang et al., 2016), they have yet to demonstrate satisfactory discriminatory power (Barichello et al., 2022). Elevated MUC1 levels show promise as predictors for the development of ARDS in sepsis patients (Wang et al., 2020). Recent research has revealed that genes associated with cuproptosis and ferroptosis, including POR, SLC7A5 and STAT3, are significantly correlated with SIC (Song et al., 2023). Machine learning algorithms have shown remarkable potential in analyzing high-dimensional data, enabling the precise identification of diagnostic and prognostic biomarkers and offering a novel approach to developing diagnostic models (Toh et al., 2019). For example, a study employing various machine learning algorithms such as elastic net, SVM, random forest and XGBoost—identified five genes capable of distinguishing between ALI and sepsis patients (Zheng et al., 2023).

Although certain biomarkers have been identified for diagnosing ARDS and SIC individually, there is a lack of systematic research on shared diagnostic markers for these conditions. Moreover, further investigation into the common pathogenic mechanisms underlying both ARDS and SIC is necessary. Proinflammatory cytokines such as TNF-α, IL-1β and IL-6 are recognized as key factors in triggering ARDS and SIC in sepsis (Kumar et al., 2007; Colás-Algora et al., 2023). Dysregulated immune responses, oxidative stress, endothelial dysfunction and coagulation abnormalities all play significant roles in the pathogenesis (Meyer et al., 2021). Therefore, an in-depth exploration of these shared mechanisms and the identification of relevant diagnostic markers hold substantial clinical implications.

In this study, we utilized bioinformatics tools, including weighted gene co-expression network analysis (WGCNA) and machine learning algorithms to identify diagnostic biomarkers and elucidate their underlying molecular mechanisms. Our objective was to discover potential diagnostic and therapeutic targets for sepsis-induced ARDS and SIC.

## Methods and materials

### Data and preprocessing

We extracted and organized data from the Gene Expression Omnibus (GEO) database (https://www.ncbi.nlm.nih.gov/geo/). The dataset GSE32707, related to sepsis-associated ARDS, includes samples from 58 sepsis patients (30 on day 0 post-admission and 28 on day 7 post-admission), 31 sepsis-induced ARDS patients (18 on day 0 and 13 on day 7 post-admission), 21 patients with systemic inflammatory response syndrome (SIRS) on day 0 and 34 control whole blood samples. We used the corresponding annotation file GPL10558 (Illumina HumanHT-12 V4.0 Expression BeadChip) to annotate the expression matrix. For our analysis, we selected sepsis-induced ARDS patients on day 0 and the control group as the discovery cohort and sepsis-induced ARDS patients on day 7 as the validation cohort. For the sepsis-induced cardiomyopathy (SIC) microarray dataset GSE79962, samples were obtained from human cardiac tissues, including 20 sepsis cardiomyopathy patients and 11 non-heart failure donor samples. GSE79962 is based on the GPL6244 platform (HuGene-1_0-st Affymetrix Human Gene 1.0 ST Array [transcript (gene) version]). GSE142615 was selected for external validation; it includes samples from mouse cardiac tissues, comprising 4 lipopolysaccharide (LPS)-induced sepsis cardiomyopathy mice and 4 saline-treated control mice. GSE142615 is based on the GPL27951 platform (Agilent-084783 *Mus musculus* array [circMouse_0410; Agilent Probe Name]). Using R version 4.3.2, we converted probes to gene symbols according to the platform annotation information. We removed non-matching probes and calculated the average expression value for genes corresponding to multiple probes.

### Identification of gene modules via weighted gene co-expression network analysis (WGCNA)

WGCNA was applied to the GSE32707 and GSE79962 datasets to identify gene modules associated with sepsis-induced ARDS and cardiomyopathy. Missing values and outliers were identified and removed through hierarchical clustering analysis. Subsequently, using the scale-free topology criterion, an appropriate “soft” threshold power (β) was determined to facilitate the construction of biologically relevant networks. A topological overlap matrix (TOM), derived from the adjacency matrix, enabled the identification of gene modules via a dynamic tree-cutting algorithm. Gene significance (GS) and module membership (MM) were calculated and their correlations with clinical characteristics were assessed, leading to the visualization of feature gene networks. Statistical measures, including the Pearson correlation coefficient and p-values of eigengenes against disease traits, were used to identify key modules closely associated with the pathogenesis of sepsis-induced ARDS and cardiomyopathy.

### Identification of differentially expressed genes (DEGs)

The identification of DEGs from the GSE32707 and GSE79962 datasets were identified using the Limma package in R (version 4.3.2), applying significance thresholds of adjusted p-value <0.05 and |log2 fold change| > 0.5. Protein–protein interaction (PPI) data and annotations were obtained from the STRING database (https://cn.string-db.org/). The analysis results were visualized through heatmaps and volcano plots, supplemented by PPI network information, with red indicating high expression and blue indicating low expression. Shared genes between the two datasets, derived from the intersection of DEGs and WGCNA module genes, were illustrated using a Venn diagram generated by jvenn (https://jvenn.toulouse.inrae.fr/app/example.html).

### Enrichment analysis

Using the clusterProfiler package in R, we analyzed Gene Ontology (GO) annotations and Kyoto Encyclopedia of Genes and Genomes (KEGG) pathways to elucidate the functional implications of the shared DEGs. Statistical significance was defined as an adjusted p-value less than 0.05.

### Leveraging machine learning for identifying diagnostic markers

We employed support vector machine recursive feature elimination (SVM-RFE) using the “e1071”, “kernlab” and “caret” packages in R for gene selection in the study of sepsis-induced conditions. Through tenfold cross-validation, this method effectively identified genes critical for disease classification, highlighting potential biomarkers. Concurrently, a random forest (RF) approach, implemented via the “Random Forest” package, was used to assess gene importance. By constructing decision trees from data subsets, this method pinpointed genes essential for predicting septic conditions and optimized the number of trees through error analysis. Gene importance was ranked based on the mean decrease in the Gini coefficient, thereby refining our selection of diagnostic markers.

Additionally, a neural network model, developed using the “neuralnet” package and visualized with “NeuralNetTools”, was employed to map gene expression to sepsis outcomes. The model featured a single hidden layer with five neurons and was trained using backpropagation to distinguish between the control and treatment groups. Model performance was evaluated by the area under the curve (AUC), revealing complex data patterns and gene interactions.

### Cell culture

Human pulmonary microvascular endothelial cells (HPMECs) were obtained from the Shanghai Biology Institute and cultured in endothelial cell medium (ScienCell, United States) supplemented with endothelial cell growth supplements (ECGS) and 5% fetal bovine serum (FBS) under a 5% CO2 atmosphere. To establish a sepsis-induced lung injury model, HPMECs were treated with lipopolysaccharide (LPS) at 10 ng/mL for 24 h, with phosphate-buffered saline (PBS) serving as the control.

### RNA extraction and quantitative Real-Time PCR (qRT‒PCR)

Total RNA was extracted using TRIzol reagent (Takara, Japan), followed by cDNA synthesis with the PrimeScript^TM^ FAST RT reagent Kit (Takara, Japan). mRNA quantification was performed by qRT-PCR using the TB Green® Premix Ex Taq^TM^ II FAST qPCR kit (Takara, Japan) on a 7,300 Real-Time PCR System (Applied Biosystems, United States). Expression levels were determined using the 2^−ΔΔCt^ method, with β-actin serving as the internal control. Results are reported as means ± standard deviations from triplicate experiments. The primer sequences utilized were as follows: SOCS3: forward: reverse: 5′-TGGTCCAGGAACTCCCGAAT-3′; β-actin: forward: AGAGCTACGAGCTGCCTGAC 5′-AGAGCTACGAGCTGCCTGAC-3′, AGCACTGTGTTGGCGTACAG 5′-AGCACTGTGTTGGCGTACAG-3′.

### Gene set enrichment analysis (GSEA)

We conducted gene set enrichment analysis (GSEA) on subgroups stratified by the median expression levels of hub genes, considering an adjusted p-value less than 0.05 as statistically significant.

Immune Cell Profiling The CIBERSORT algorithm was employed to assess immune cell composition by estimating the relative proportions of 22 immune cell types from gene expression data. Samples with a CIBERSORT p-value less than 0.05 were included and the output estimates were normalized to enable comparisons across datasets. Visualization was performed using R packages and Spearman correlation analysis was conducted to determine associations between infiltrating immune cells and diagnostic biomarkers.

### Building the ceRNA network and mapping TF‒Gene interactions

To dissect the regulatory competing endogenous RNA (ceRNA) network surrounding our hub genes, we integrated miRNA target predictions from miRDB (https://mirdb.org/), TargetScan (https://www.targetscan.org/vert_80/) and miRanda (http://www.microrna.org/) to identify targeted miRNAs, emphasizing accuracy through cross-database consensus. SpongeScan (http://spongescan.rc.ufl.edu/) was employed to identify long non-coding RNAs (lncRNAs) potentially acting as miRNA sponges. Additionally, NetworkAnalyst (https://www.networkanalyst.ca/) was utilized to identify transcription factors (TFs) associated with our hub genes. These predicted interaction networks were effectively visualized using Cytoscape version 3.8.2.

### Predicting drug candidates and molecular docking

We sourced small molecules that interact with target genes from the CTD database (http://ctdbase.org/) and performed molecular docking using these molecules as ligands and the central genes as receptors. The 3D structures of the ligands were downloaded in SDF format from PubChem and processed using Chem3D 19.0, then saved in mol2 format for compatibility. The target proteins were modeled via SwissModel (https://swissmodel.expasy.org/) and their structures validated using SAVES v6.0 (https://saves.mbi.ucla.edu/). Molecular docking was conducted in MOE 2019, with PyMOL 2.5 employed to visualize the interactions. Binding affinity, indicated by free energy values, was used to guide our analysis: values below −1.2 kcal/mol suggested activity, while those below −5.0 kcal/mol indicated strong affinity, providing potential therapeutic insights.

### Statistical analysis

Statistical analysis of the data from this study was performed using R (version 4.3.2). A t-test was conducted to compare continuous variables between two groups, assuming a normal distribution. To assess the correlation between gene expression and immune cell fractions, the Spearman rank correlation test was employed. The qPCR results were analyzed using a t-test with the statistical software SPSS version 23.0. Statistical significance was defined as a p-value less than 0.05.

## Results

### Screening key modules through WGCNA

**FIGURE 1:**
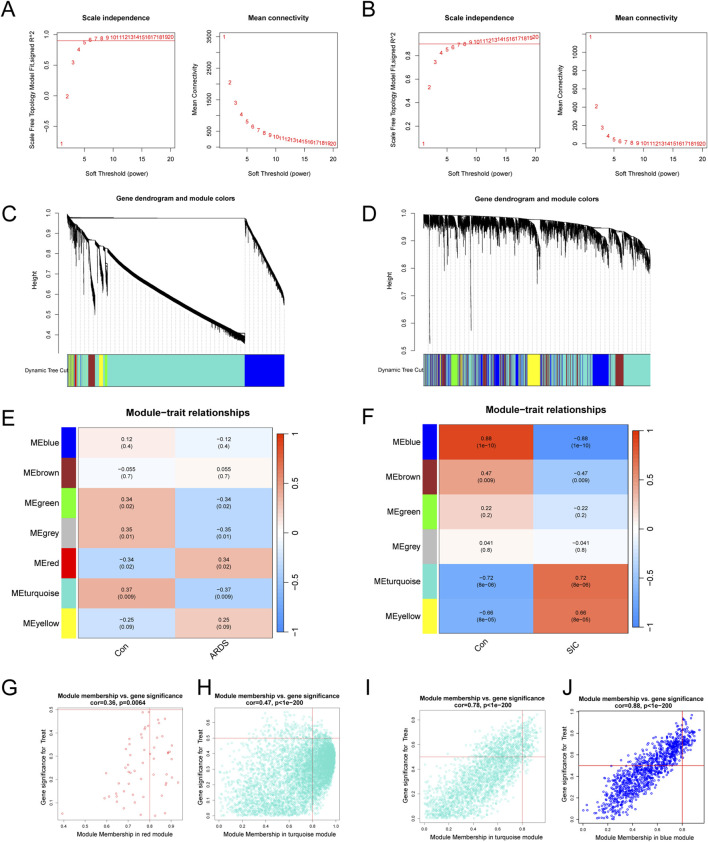
Analysis and identification of modules in the weighted gene co-expression network (WGCNA). **(A)** Determination of the soft-thresholding power for WGCNA in ARDS. **(B)** Calculation of the soft-thresholding power for SIC. **(C)** Cluster dendrogram for ARDS, highlighting key modules of highly connected genes. **(D)** Cluster dendrogram for SIC, showing modules with highly connected genes. **(E)** Module‒trait relationships in ARDS, including correlation coefficients and p-values for each module. **(F)** Module‒trait relationships in SIC, detailing correlations and p-values for each module. **(G)** Scatter plots illustrating gene significance (GS) versus module membership (MM) for genes within the red modules in ARDS. **(H)** Scatter plots illustrating gene significance (GS) versus module membership (MM) for genes within the turquoise modules in ARDS. **(I)** Scatter plots showing the GS against MM for genes in the turquoise modules in SIC. **(J)** Scatter plots showing the GS against MM for genes in the blue modules in SIC.

Weighted gene co-expression network analysis (WGCNA) was employed to identify co-expressed and variably expressed gene modules in patients with sepsis-induced ARDS and SIC, and to analyze their associations with disease spectra. For sepsis-induced ARDS patients, a soft-thresholding power of 5 was selected (Figure 1A). An adjacency matrix was generated using the adjacency function, followed by hierarchical clustering based on TOM dissimilarity, which revealed seven co-expression modules (Figure 1C). Notably, modules with P < 0.05 were highlighted as key modules (Figure 1E), with the red module showing the most significant positive correlation (56 genes, r = 0.34, p = 0.02), while the turquoise module exhibited the most significant negative correlation (6,223 genes, r = −0.37, p = 0.009). Additionally, significant associations were found between module membership and gene significance in the red module (r = 0.36, p = 0.0064) and the turquoise module (r = 0.47, p < 1e-200), as shown in Figures 1G,H, respectively. Consequently, 6,279 key genes significantly associated with sepsis-induced ARDS were identified in the red and turquoise modules.

For the SIC analysis, a soft-thresholding power (β) of 5 was determined as optimal (Figure 1B), resulting in the identification of six modules. The turquoise module exhibited a strong positive correlation (2,450 genes, r = 0.72, p = 8e−6), while the blue module exhibited a significant negative correlation (1,049 genes, r = −0.88, p = 1e−10, Figures 1D,F). Additionally, significant correlations were observed between gene significance (GS) and module membership (MM) within both the turquoise and blue modules. The turquoise module demonstrated a correlation coefficient of 0.78 (p < 1e-200, Figure 1I) and the blue module displayed a correlation coefficient of 0.88 (p < 1e-200, Figure 1J). In total, 3,499 key genes significantly associated with SIC were identified across the turquoise and blue modules.

### Unveiling and investigating shared differentially expressed genes (DEGs)

**FIGURE 2:**
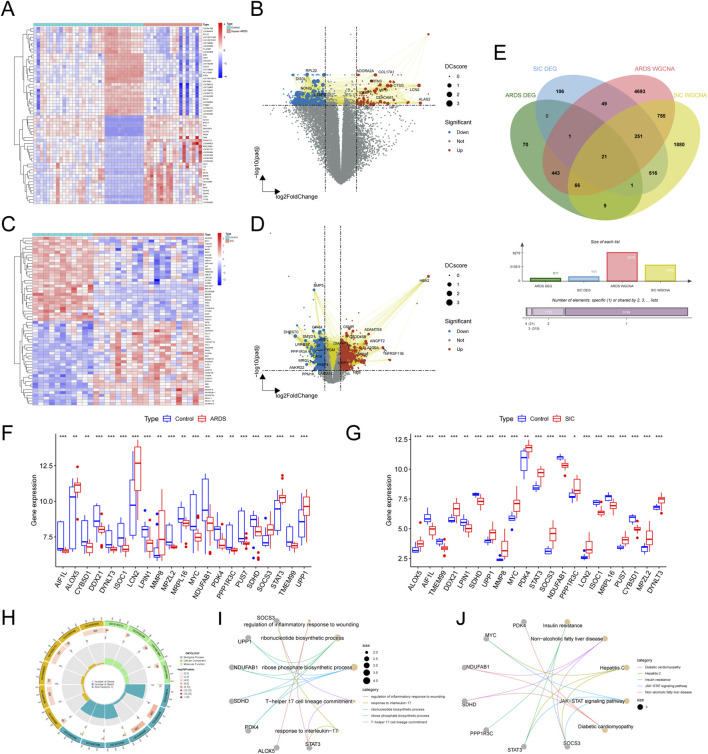
Exploring shared DEGs and their functional significance. **(A)** Heatmap of DEGs in the GSE32707-day0 dataset. **(B)** Volcano plot of DEGs in the GSE32707-day0 dataset. **(C)** Heatmap of DEGs in the GSE79962 dataset. **(D)** Volcano plot of DEGs in the GSE79962 dataset. **(E)** Venn diagram highlighting 21 key shared genes identified from the DEGs and network modules. **(F)** Boxplots showing expression variations of the 21 shared genes in the ARDS and SIC groups compared to the normal group. **(G)** Boxplots showing expression variations of the 21 shared genes in the ARDS and SIC groups compared to the normal group. **(H)** Circle chart illustrating the results of the GO enrichment analysis. **(I)** Network graph illustrating the results of the GO enrichment analysis. **(J)** Network graph depicting KEGG pathway enrichment results.

Our comparative study of patients with sepsis-related ARDS and healthy individuals identified 611 differentially expressed genes (DEGs), of which 114 were upregulated and 497 downregulated (Figure 2A). These DEGs were visualized in a protein–protein interaction (PPI) integrated volcano plot (Figure 2B) and the 30 most significant DEGs were further highlighted in a heatmap (Figure 2A). In the comparison between SIC and the control group, 945 DEGs were identified, including 481 upregulated and 464 downregulated genes, with particular emphasis on the top 30 genes shown in Figures 2C,D. By intersecting DEGs with genes from the WGCNA modules, 21 shared genes were identified for in-depth investigation (Figure 2E), suggesting their potential as biomarkers for the pathogenesis of sepsis-induced ARDS and SIC. The expression levels of these genes in the two disease groups and the control group are illustrated in Figures 2F,G.

To elucidate the underlying mechanisms, functional enrichment analysis was carried out through GO and KEGG pathway analyses. The shared genes were associated with biological processes (BP) related to inflammation and immunity, including the regulation of the inflammatory response, ribonucleotide biosynthesis and T-helper 17 cell lineage commitment. These genes are linked to specific cellular components (CC) and molecular functions (MF) (Figures 2F–I). KEGG pathway analysis highlighted the significant involvement of these genes in the JAK-STAT signaling pathway (Figure 2J).

### Identifying diagnostic biomarkers through machine learning algorithms

**FIGURE 3:**
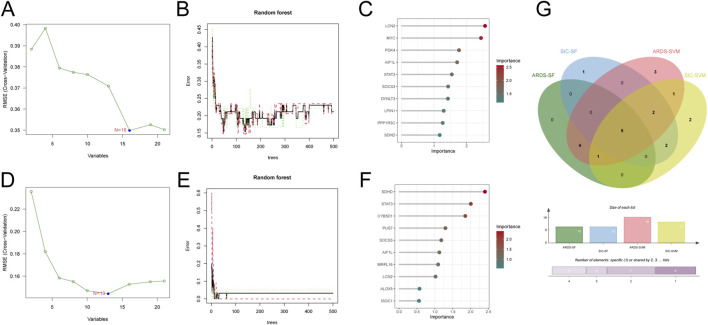
Machine learning identifies diagnostic genes for sepsis-induced ARDS and SIC. **(A)** Screening of ARDS crosstalk genes with SVM-RFE. **(B)** Accuracy of the random forest model for ARDS, shown with error rate confidence intervals. **(C)** Top 10 ARDS genes ranked by discriminative power in the random forest model. **(D)** Identification of SIC crosstalk genes with SVM-RFE. **(E)** Accuracy of the random forest model for SIC, depicted with error rate confidence intervals. **(F)** Top 10 discriminative SIC genes in the random forest model. **(G)** Venn diagram illustrating the five common diagnostic genes identified in both ARDS and SIC patients through machine learning analysis.

To identify diagnostic biomarkers for sepsis-induced ARDS and SIC, we applied two machine learning methods - support vector machine (SVM) and random forest (RF). Using SVM, we identified 16 crucial diagnostic genes for ARDS (Figures 3A,B). With RF, we evaluated gene importance and highlighted the top 10 most significant genes (Figure 3C). Additionally, by employing SVM regression and RF, we discovered 13 and 10 core diagnostic genes for SIC, respectively (Figures 3D–F). Comparing the results from both conditions using these machine learning techniques, we identified five shared diagnostic genes: LCN2, AIF1L, STAT3, SOCS3 and SDHD (Figure 3G).

### Building an artificial neural network (ANN) model for diagnosis

**FIGURE 4:**
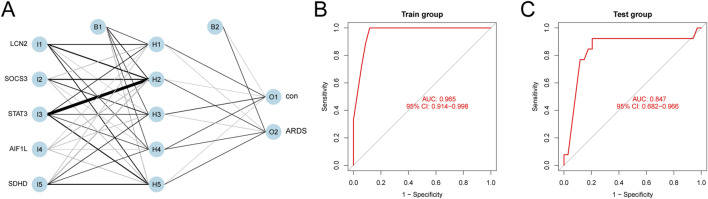
ANN Model Diagnostics. **(A)** Ability of the ANN model to differentiate between ARDS patients and healthy subjects. **(B)** ROC curve of the model using the training dataset. **(C)** ROC curve from the evaluation of the testing dataset.

We developed an artificial neural network (ANN) model using five characteristic biomarkers. The results demonstrate its strong ability to distinguish between ARDS patients and healthy individuals (Figure 4A; supplementary material: ANN model). Furthermore, ROC curves were employed to evaluate the overall diagnostic performance of the ANN model. In the training set, the model achieved an AUC of 0.965 (95% CI: 0.914–0.998), indicating excellent discrimination (Figure 4B). Similarly, in the testing set, it demonstrated positive diagnostic performance, with an AUC of 0.847 (95% CI: 0.682–0.966) (Figure 4C).

### Confirmation and validation of diagnostic marker genes

**FIGURE 5:**
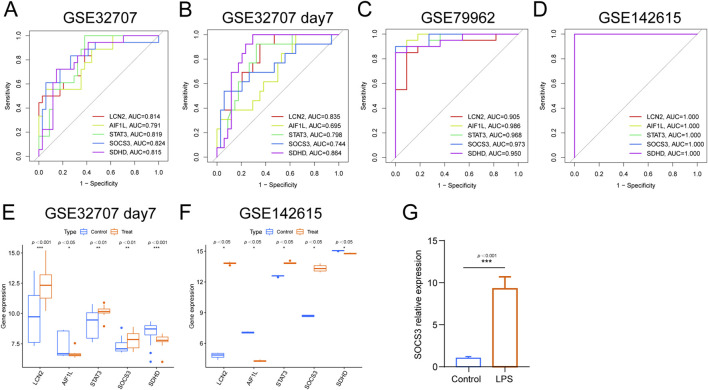
Prediction efficiency of the intersected genes. **(A)** ROC curves for LCN2, AIF1L, STAT3, SOCS3 and SDHD in GSE32707 Day 0, **(B)** ROC curves for LCN2, AIF1L, STAT3, SOCS3 and SDHD in GSE32707 Day 7. **(C)** ROC curves for LCN2, AIF1L, STAT3, SOCS3 and SDHD in GSE79962. **(D)** ROC curves for LCN2, AIF1L, STAT3, SOCS3 and SDHD in GSE142615. **(E)** Expression profiles of LCN2, AIF1L, STAT3, SOCS3 and SDHD in the training cohorts GSE32707 Day 7. **(F)** Expression profiles of LCN2, AIF1L, STAT3, SOCS3 and SDHD in the training cohorts GSE142615. **(G)** qPCR analysis of SOCS3 expression in HPMECs.

We investigated the diagnostic utility of five genes in sepsis-related ARDS and SIC using both training and testing cohorts. In ARDS samples, ROC analysis of the training cohort yielded AUC-ROC values for LCN2, AIF1L, STAT3, SOCS3 and SDHD of 0.814, 0.791, 0.819, 0.824 and 0.815, respectively (Figure 5A). When GSE72707 DAY 7 was used as the test set, the AUCs of these genes were 0.835, 0.695, 0.798, 0.744 and 0.864, respectively (Figure 5B). For the SIC samples, the AUC values in the training cohort ranged from 0.905 to 0.986 (Figure 5C) and reached a perfect score of 1 in the external test set, GSE142615 (Figure 5D), highlighting their strong diagnostic potential.

Additionally, a comparative analysis of the five characteristic biomarkers was conducted. In the test set, the expression profiles of LCN2, STAT3 and SOCS3 notably increased, whereas those of AIF1L and SDHD decreased in both ARDS and SIC groups, consistent with the findings from the training set (Figure 5E). Using qPCR, we further validated SOCS3 expression and found that SOCS3 levels were significantly elevated in LPS-treated HPMECs in the sepsis-induced lung injury model group compared to the control group.

### Establishing a septic injury model to investigate SOCS3 expression

We observed that SOCS3 demonstrated strong and consistent diagnostic potential. To further explore its expression and functional roles and to establish a foundation for future mechanistic studies, we developed a septic injury model in human pulmonary microvascular endothelial cells (HPMECs) using lipopolysaccharide (LPS) and assessed SOCS3 expression. The results revealed a marked increase in SOCS3 expression in the LPS-treated group compared to the control group (P < 0.05), consistent with our bioinformatics findings (Figure 5G).

### GSEA of SOCS3 and analysis of immune infiltration

**FIGURE 6:**
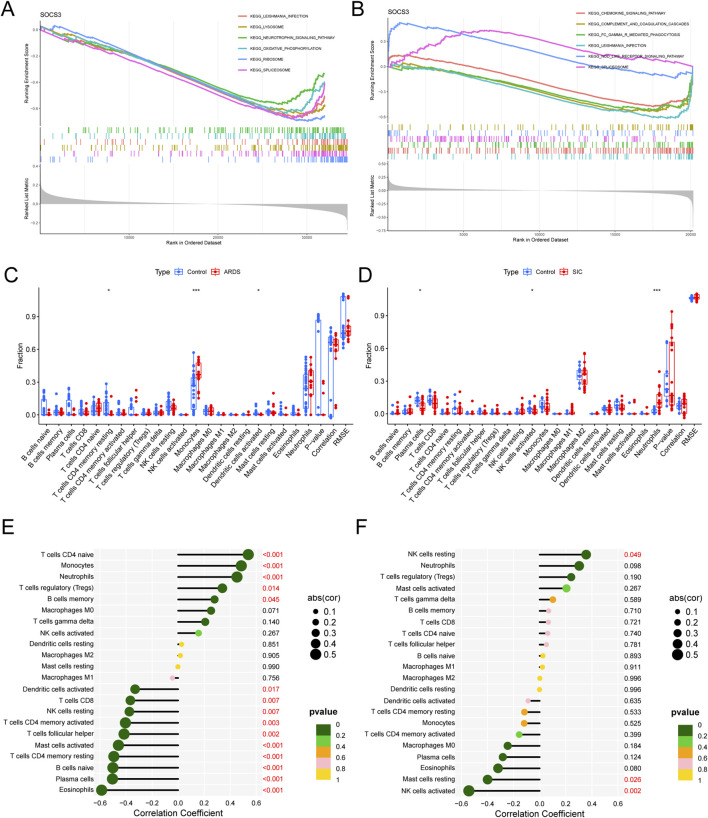
GSEA of hub genes and analysis of immune infiltration. **(A)** GSEA of SOCS3 in ARDS. **(B)** GSEA of SOCS3 in SIC. **(C)** Comparison of immune cell infiltration between ARDS patients and normal controls. **(D)** Comparison of immune cell infiltration between SIC patients and normal controls. **(E)** Association between SOCS3 expression and infiltration of different immune cells in ARDS. **(F)** Association between SOCS3 expression and infiltration of different immune cells in SIC. *p < 0.05; **P < 0.01; ***P < 0.001.

We performed single-gene gene set enrichment analysis (GSEA) on the SOCS3 gene within the ARDS and SIC datasets. The analysis revealed strong associations between SOCS3 and several key pathways. In ARDS, these pathways included the ribosome, spliceosome, lysosome and oxidative phosphorylation pathways (Figure 6A). In contrast, in SIC, significant connections were observed with the chemokine signaling pathway, Fc gamma receptor-mediated phagocytosis, the NOD-like receptor signaling pathway and the complement and coagulation cascades (Figure 6B).

Given the pivotal role of immune and inflammatory reactions, we utilized the CIBERSORT algorithm for our analysis. Our findings revealed an increase in monocytes and a decrease in CD4 memory resting T cells and activated dendritic cells in ARDS patients (Figure 6C), whereas neutrophil counts increased and plasma cells and activated NK cells decreased in SIC patients (Figure 6D).

Moreover, in ARDS, SOCS3 expression was positively correlated with CD4^+^ naive T cells, monocytes, neutrophils and regulatory T cells (Tregs) and negatively correlated with activated mast cells, CD4^+^ memory resting T cells, naive B cells and plasma cells (Figure 6E). In the context of SIC, SOCS3 was positively correlated with resting NK cells but inversely linked with resting mast cells and activated NK cells (Figure 6F), emphasizing the essential role of immune mechanisms in the progression of these conditions.

### Prediction of the ceRNA network and transcription factor interactions targeting SOCS3

**FIGURE 7:**
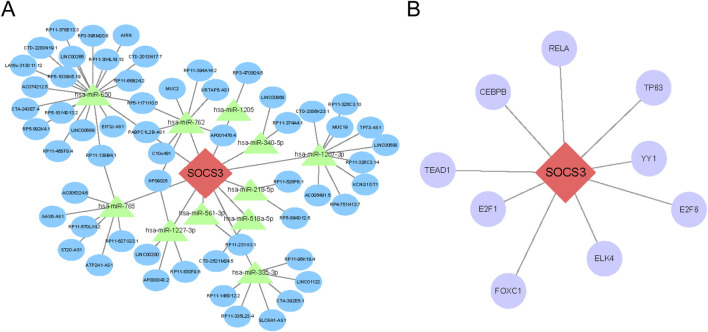
Prediction of the ceRNA network and transcription factor (TF) interactions that target SOCS3. **(A)** Construction of the lncRNA‒miRNA-mRNA ceRNA network involving SOCS3. **(B)** Regulatory interaction network illustrating connections between SOCS3 and TFs. Hub genes are depicted in red‒orange, miRNAs in green, lncRNAs in blue and TFs in purple.

Using relevant databases, we predicted interactions within the ceRNA network and identified transcription factors (TFs) that target SOCS3. As illustrated in Figure 7A, we constructed a regulatory network comprising 69 nodes—SOCS3, 11 miRNAs, and 57 lncRNAs—and 71 edges. Additionally, a TF‒mRNA network was established (Figure 7B), highlighting 9 transcription factors that target SOCS3.

### Potential drug prediction and molecular docking analysis

**FIGURE 8:**
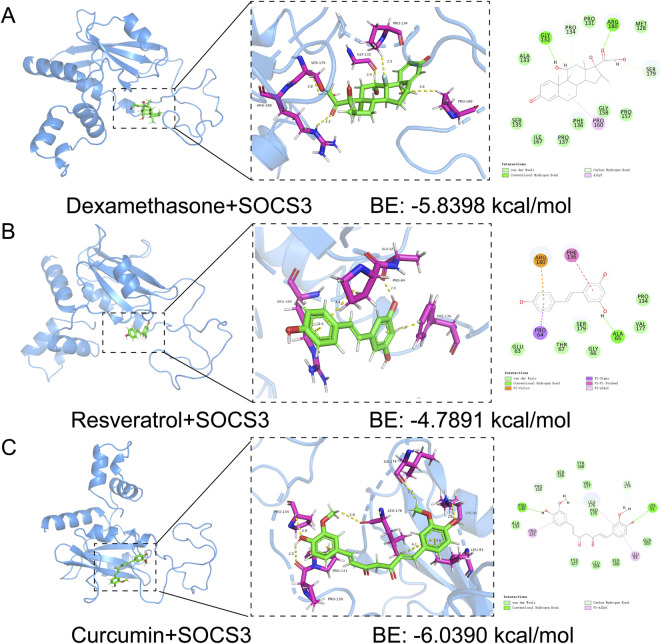
Molecular docking of dexamethasone. **(A)** Resveratrol. **(B)** Curcumin. **(C)** With SOCS3.

Our study utilized molecular docking to identify potential therapeutic drugs. By screening the PubChem database, we focused on dexamethasone, resveratrol and curcumin. Docking simulations revealed favorable binding affinities between these compounds and the SOCS3 protein, with binding energies below −1.2 kcal/mol. Specifically, dexamethasone, resveratrol and curcumin presented binding energies of −5.8398 kcal/mol, −4.7891 kcal/mol and −6.0390 kcal/mol, respectively (Figures 8A–C). These results highlight the potential of these compounds to target SOCS3, supporting further investigation and development for treating sepsis-associated lung injury and cardiomyopathy.

## Discussion

Sepsis-induced acute respiratory distress syndrome (ARDS) and sepsis-induced cardiomyopathy (SIC) are two grave complications of sepsis that are linked to high mortality rates. These conditions stem from an overactive inflammatory response that cause widespread tissue and organ damage. In sepsis, inflammatory mediators can simultaneously trigger ARDS and impair cardiac function, leading to cardiomyopathy, thereby complicating ARDS management (Li et al., 2019; Zhang and Ning, 2021). Using bioinformatics methods, we screened out the genes shared by ARDS and SIC patients and analyzed their pathways, revealing significant enrichment in immune and inflammatory pathways. Effective treatment of these complications requires rapid and precise diagnosis. Although the SIC signature was originally derived from cardiac tissue, which is not a feasible source for routine clinical diagnostics, the concomitant discovery of these same biomarkers in the readily accessible peripheral blood of ARDS patients provides a clear and practical path for translational application. Our work suggests that a blood-based test for this gene signature could non-invasively report on the status of distant organ dysfunction, a concept that warrants vigorous future validation. A thorough understanding of the shared pathophysiological mechanisms between ARDS and SIC is crucial for developing effective therapies and improving patient outcomes. Our study not only advances the current knowledge of the pathogenesis of ARDS and SIC but also identifies potential diagnostic biomarkers and therapeutic targets, opening new avenues for research and clinical intervention.

Sepsis pathogenesis involves a highly intricate process characterized by dysregulated inflammatory responses, immune system dysfunction, mitochondrial damage, coagulation disorders, abnormalities in the neuroendocrine immune network, endoplasmic reticulum stress, autophagy and various other pathophysiological mechanisms. These factors collectively contribute to organ dysfunction (Zhang et al., 2023).

The application of machine learning algorithms to identify diagnostic biomarkers represents an innovative approach, bridging the gap between genomic data and clinical practice (Handelman et al., 2018). We utilized weighted gene co-expression network analysis (WGCNA) and machine learning algorithms to identify shared diagnostic markers potentially linked to sepsis-induced acute respiratory distress syndrome (ARDS) and cardiomyopathy. Our findings indicate that biomarkers such as LCN2, AIF1L, STAT3, SOCS3 and SDHD have significant potential for the early detection and prognosis of ARDS and SIC. Furthermore, the development of an artificial neural network (ANN) model for diagnosis marks a promising advancement in precision medicine; however, integrating this technology into clinical practice will require overcoming substantial technological and regulatory challenges. Notably, these promising biomarkers must undergo rigorous validation in larger and more diverse patient cohorts before clinical application.

Our study has three key limitations. First, as a discovery-phase investigation, it focused on the computational identification of novel shared biomarkers rather than clinical validation, leaving critical parameters (e.g., cut-off values and dose-effect relationships) for future multi-center prospective studies. Second, the cross-sectional design of the datasets (GSE32707, GSE79962), combined with inherent heterogeneity in sepsis etiology and limited sample sizes, restricts our ability to analyze temporal biomarker dynamics or their correlation with disease progression. Primarily, the lack of clinical feature data (e.g., age, gender, comorbidities) in these genomic datasets hinders the integration of model predictions with clinical factors. Third, although we identified shared molecular pathways (e.g., those involving SOCS3) associated with sepsis-induced organ damage, the underlying biological mechanisms through which dysregulation of these pathways directly drives tissue injury remain poorly understood and require further functional validation (e.g., via *in vitro* cell models or animal studies). Addressing these gaps is indispensable for translating our computational findings into actionable clinical tools and advancing targeted therapeutic strategies.

LCN2, also known as neutrophil gelatinase-associated lipocalin (NGAL), serves as a key inflammatory marker closely linked to infection and inflammation. Research indicates that LCN2 plays a significant role in inflammation and oxidative stress in acute lung injury (Wang et al., 2022). It has been identified as a potential therapeutic target for conditions such as pneumonia and acute lung injury (An et al., 2023). Allograft inflammatory factor 1-like (AIF1L) is expressed primarily in immune cells and may contribute to regulating the inflammatory response (Yasuda-Yamahara et al., 2018). Recent research has revealed that lactate inhibits T cell activation by reducing the expression of CD40LG and suppressing the SOCS3-mediated JAK1/STAT3 signaling pathway (Zhang et al., 2025a). STAT3 and SOCS3 are critical regulators of inflammatory signaling pathways (Wang et al., 2024). STAT3 functions as both a signaling activator and a transcriptional activator, influencing cellular proliferation, differentiation and apoptosis. A recent study demonstrated that verapamil and tangeretin confer protection against lipopolysaccharide (LPS)-induced sepsis by reducing the population of M1 macrophages. This protective effect is also mediated through the inhibition of P-glycoprotein expression, achieved by the downregulation of STAT1 and STAT3 signaling pathways alongside the upregulation of SOCS3 expression within macrophages (Kundu et al., 2024). In recent years, increasing evidence indicates that severe sepsis causes mitochondrial structural damage in heart muscle cells, including apoptosis, incomplete autophagy, and mitophagy and impairs their function, resulting in ATP depletion (Kuroshima et al., 2024). SDHD is a subunit of the mitochondrial respiratory chain complex II, which is crucial for cellular energy metabolism and the management of oxidative stress (Lin et al., 2021; Han et al., 2023). Dysregulated expression of SDHD can lead to mitochondrial dysfunction and heightened oxidative stress, potentially contributing to tissue damage in the heart and lungs (Bejarano-García et al., 2016; Zhao et al., 2021).

SOCS3 functions as a negative regulator by controlling immune and inflammatory responses through the inhibition of cytokine signaling pathways, particularly the JAK/STAT3 pathway, which is essential for cytokine signal transduction (Gao et al., 2018). Research has shown that ADAR1 targets miR-30a to regulate SOCS3 expression, thereby reducing IL-6 levels, mitigating inflammation and organ damage and providing protection against sepsis (Shangxun et al., 2020). In sepsis-induced ARDS, dysregulated inflammation is a major contributor to lung injury and hypoxemia, with SOCS3 potentially influencing the occurrence and progression of ARDS by modulating inflammatory signaling pathways (Sun et al., 2024). Similarly, in sepsis-induced cardiomyopathy, SOCS3 may participate in pathological processes by regulating cardiomyocyte growth, apoptosis and immune response modulation. Feng et al. established a septic cardiomyopathy (SCM) model by stimulating HL-1 and AC16 cells with LPS, which resulted in a significant increase in SOCS3 expression (Lu et al., 2022). Weng demonstrated that the neutralization of interleukin-33 (IL-33) attenuates inflammation, oxidative stress, and apoptosis in cardiomyocytes through the modulation of the nuclear factor kappa B (NF-κB), signal transducer and activator of transcription 3 (STAT3), and suppressors of cytokine signaling 3 (SOCS3) signaling pathway (Weng et al., 2025). Therefore, in this study, we utilized LPS stimulation of only human pulmonary microvascular endothelial cells (HPMECs) to construct a sepsis-induced lung injury model in which a notable increase in SOCS3 expression was observed. The role of SOCS3 in inflammation and immune regulation may be critical for ARDS and myocardial disease induced by sepsis. By regulating the inflammatory signaling pathway and immune response, SOCS3 may influence the onset, progression, and severity of these conditions, offering new targets and strategies for their treatment.

The discovery of these genes opens new avenues for diagnosing and treating sepsis-induced ARDS and cardiomyopathy. However, further clinical research and validation are necessary to assess their feasibility and effectiveness in clinical settings. The discrepancies between our findings and those of previous studies pose intriguing questions about the variability in sepsis pathophysiology. For example, while our study highlights the significant role of the SOCS3 gene in both ARDS and SIC—a gene that has been relatively understudied in the literature—this underscores the complexity of sepsis and its complications. Factors such as genetic background, environmental influences and the nature of the initiating infection may profoundly influence disease outcomes. Exploring the potential long-term and latent effects of these identified biomarkers and therapeutic targets is essential. Additionally, the interaction between these genetic factors and other determinants of sepsis severity, such as comorbidities and treatment interventions, remains an important area for further investigation.

We performed targeted screening of the diagnostic gene SOCS3 to develop a novel therapeutic approach for reducing organ damage in sepsis. Dexamethasone, a corticosteroid widely used for treating inflammatory diseases, significantly reduces mortality in early-stage moderate-to-severe ARDS patients (Villar et al., 2020). However, high systemic doses can lead to severe side effects such as hyperglycemia, peptic ulcers and electrolyte imbalances (Madamsetty et al., 2022). Resveratrol, an antioxidant and anti-inflammatory polyphenol, reduces oxidative stress and inflammation, thereby improving lung function and cardiac protection (Meng et al., 2020). Resveratrol also inhibited CD45+Siglec F− and CD45^+^CD206− M1 subtype macrophages via the SOCS3 signaling pathway in a murine model of LPS-induced acute lung injury (ALI) (Hu et al., 2019). A self-assembling nanopeptide and resveratrol hydrogel composite enhances Sirt1-mediated deacetylation of p62, promoting mitochondrial autophagy and immunometabolic remodeling, thereby mitigating sepsis-induced inflammation (Wang et al., 2025). Curcumin has the capacity to mitigate the cytokine storm and reduce organ damage induced by sepsis through various mechanisms. These include its anti-inflammatory and antioxidant properties, suppression of inflammatory cell death, protection of vascular endothelial integrity, maintenance of mitochondrial function and regulation of immune cell activity (Zhang et al., 2025b). Curcumin, known for its potent anti-inflammatory effects, acts on the NF-κB, MAPK, AP-1 and Jak/STAT pathways but faces pharmacokinetic challenges that limit its clinical use (Luo et al., 2025). Current research focuses on curcumin derivatives, prodrugs and combination therapies to enhance its delivery and efficacy (Peng et al., 2021). These three compounds have demonstrated high-affinity binding to SOCS3 and their combined application is expected to amplify therapeutic benefits while minimizing side effects. Overall, our findings highlight dexamethasone, resveratrol and curcumin as promising SOCS3-targeting agents for treating sepsis-induced lung injury and cardiomyopathy, paving the way for further *in vitro*, *in vivo* and clinical development.

Our study provides valuable insights into the molecular basis of ARDS and SIC in the context of sepsis, identifying novel biomarkers and pathways for potential therapeutic intervention. While we have made significant progress in understanding these complex conditions, our work also reveals the vast array of unknowns that remain. Moving forward, a multidisciplinary approach integrating advanced genomic analysis, bioinformatics and clinical research will be essential to fully unravel the complexities of sepsis and its devastating complications. As we explore this uncharted territory, our findings lay a foundation for future studies aimed at improving outcomes for patients with ARDS and SIC.

## Data Availability

The datasets presented in this study can be found in online repositories. The names of the repository/repositories and accession number(s) can be found in the article/supplementary material.

## Abbreviations

ARDS, acute respiratory distress syndrome; SIC, sepsis-induced cardiomyopathy; DEGs, differentially expressed genes; WGCNA, weighted gene co-expression network analysis; SVM-RFE, support vector machine-recursive feature elimination; RF, random forest; ANN, artificial neural network; CIBERSORT, cell type proportions from RNA transcripts; GSEA, gene set enrichment analysis; ALI, acute lung injury.

## References

[B1] An H. S. Yoo J. W. Jeong J. H. Heo M. Hwang S. H. Jang H. M. (2023). Lipocalin-2 promotes acute lung inflammation and oxidative stress by enhancing macrophage iron accumulation. Int. J. Biol. Sci. 19, 1163–1177. 10.7150/ijbs.79915 36923935 PMC10008694

[B2] Barichello T. Generoso J. S. Singer M. Dal-Pizzol F. (2022). Biomarkers for sepsis: more than just fever and leukocytosis-a narrative review. Crit. Care 26, 14. 10.1186/S13054-021-03862-5 34991675 PMC8740483

[B3] Bejarano-García J. A. Millán-Uclés Á. Rosado I. V. Sánchez-Abarca L. I. Caballero-Velázquez T. Durán-Galván M. J. (2016). Sensitivity of hematopoietic stem cells to mitochondrial dysfunction by SdhD gene deletion. Cell Death Dis. 7, e2516. 10.1038/cddis.2016.411 27929539 PMC5261010

[B4] Colás-Algora N. Muñoz-Pinillos P. Cacho-Navas C. Avendaño-Ortiz J. De Rivas G. Barroso S. (2023). Simultaneous targeting of IL-1-Signaling and IL-6-Trans-Signaling preserves human pulmonary endothelial barrier function during a cytokine storm-brief report. Arterioscler. Thromb. Vasc. Biol. 43, 2213–2222. 10.1161/ATVBAHA.123.319695 37732482

[B5] Coopersmith C. M. De Backer D. Deutschman C. S. Ferrer R. Lat I. Machado F. R. (2018). Surviving sepsis campaign: research priorities for sepsis and septic shock. Intensive Care Med. 44, 1400–1426. 10.1007/S00134-018-5175-Z 29971592 PMC7095388

[B6] Gao Y. Zhao H. Wang P. Wang J. Zou L. (2018). The roles of SOCS3 and STAT3 in bacterial infection and inflammatory diseases. Scand. J. Immunol. 88, e12727. 10.1111/sji.12727 30341772

[B7] Gorman E. A. O’Kane C. M. McAuley D. F. (2022). Acute respiratory distress syndrome in adults: diagnosis, outcomes, long-term sequelae, and management. Lancet 400, 1157–1170. 10.1016/S0140-6736(22)01439-8 36070788

[B8] Han S. H. Lee M. Shin Y. Giovanni R. Chakrabarty R. P. Herrerias M. M. (2023). Mitochondrial integrated stress response controls lung epithelial cell fate. Nature 620, 890–897. 10.1038/s41586-023-06423-8 37558881 PMC10447247

[B9] Handelman G. S. Kok H. K. Chandra R. V. Razavi A. H. Lee M. J. Asadi H. (2018). eDoctor: machine learning and the future of medicine. J. Intern Med. 284, 603–619. 10.1111/joim.12822 30102808

[B10] Hu L. Chen Z. Li L. Jiang Z. Zhu L. (2019). Resveratrol decreases CD45+CD206− subtype macrophages in LPS-induced murine acute lung injury by SOCS3 signalling pathway. J. Cell Mol. Med. 23, 8101–8113. 10.1111/jcmm.14680 31559687 PMC6850919

[B11] Kumar A. Kumar A. Paladugu B. Mensing J. Parrillo J. E. (2007). Transforming growth factor-beta1 blocks *in vitro* cardiac myocyte depression induced by tumor necrosis factor-alpha, interleukin-1beta, and human septic shock serum. Crit. Care Med. 35, 358–364. 10.1097/01.CCM.0000254341.87098.A4 17204997

[B12] Kundu A. Ghosh P. Bishayi B. (2024). Verapamil and tangeretin enhances the M1 macrophages to M2 type in lipopolysaccharide-treated mice and inhibits the P-glycoprotein expression by downregulating STAT1/STAT3 and upregulating SOCS3. Int. Immunopharmacol. 133, 112153. 10.1016/J.INTIMP.2024.112153 38678669

[B13] Kuroshima T. Kawaguchi S. Okada M. (2024). Current perspectives of mitochondria in sepsis-induced cardiomyopathy. Int. J. Mol. Sci. 25, 4710. 10.3390/IJMS25094710 38731929 PMC11083471

[B14] Li N. Zhou H. Wu H. Wu Q. Duan M. Deng W. (2019). STING-IRF3 contributes to lipopolysaccharide-induced cardiac dysfunction, inflammation, apoptosis and pyroptosis by activating NLRP3. Redox Biol. 24, 101215. 10.1016/J.REDOX.2019.101215 31121492 PMC6529775

[B15] Lin S. Fasham J. Al-Hijawi F. Qutob N. Gunning A. Leslie J. S. (2021). Consolidating biallelic SDHD variants as a cause of mitochondrial complex II deficiency. Eur. J. Hum. Genet. 29, 1570–1576. 10.1038/s41431-021-00887-w 34012134 PMC8484551

[B16] Lu F. Hu F. Qiu B. Zou H. Xu J. (2022). Identification of novel biomarkers in septic cardiomyopathy via integrated bioinformatics analysis and experimental validation. Front. Genet. 13, 929293. 10.3389/fgene.2022.929293 35957694 PMC9358039

[B17] Luo J. Luo J. Fang Z. Fu Y. Xu B. (2025). Insights into effects of natural bioactive components on inflammatory diseases in respiratory tract. Phytother. Res. 39, 4199–4229. 10.1002/PTR.8367 40767628 PMC12423489

[B18] Madamsetty V. S. Mohammadinejad R. Uzieliene I. Nabavi N. Dehshahri A. García-Couce J. (2022). Dexamethasone: insights into pharmacological aspects, therapeutic mechanisms, and delivery systems. ACS Biomater. Sci. Eng. 8, 1763–1790. 10.1021/acsbiomaterials.2c00026 35439408

[B19] Martin L. Derwall M. Al Zoubi S. Zechendorf E. Reuter D. A. Thiemermann C. (2019). The septic heart: current understanding of molecular mechanisms and clinical implications. Chest 155, 427–437. 10.1016/J.CHEST.2018.08.1037 30171861

[B20] Meng X. Zhou J. Zhao C. N. Gan R. Y. Li H. B. (2020). Health benefits and molecular mechanisms of resveratrol: a narrative review. Foods 9, 340. 10.3390/foods9030340 32183376 PMC7143620

[B21] Meyer N. J. Gattinoni L. Calfee C. S. (2021). Acute respiratory distress syndrome. Lancet 398, 622–637. 10.1016/S0140-6736(21)00439-6 34217425 PMC8248927

[B22] Peng Y. Ao M. Dong B. Jiang Y. Yu L. Chen Z. (2021). Anti-inflammatory effects of curcumin in the inflammatory diseases: status, limitations and countermeasures. Drug Des. Devel Ther. 15, 4503–4525. 10.2147/DDDT.S327378 34754179 PMC8572027

[B23] Rudd K. E. Johnson S. C. Agesa K. M. Shackelford K. A. Tsoi D. Kievlan D. R. (2020). Global, regional, and national sepsis incidence and mortality, 1990-2017: analysis for the global burden of disease study. Lancet 395, 200–211. 10.1016/S0140-6736(19)32989-7 31954465 PMC6970225

[B24] Shangxun Z. Junjie L. Wei Z. Yutong W. Wenyuan J. Shanshou L. (2020). ADAR1 alleviates inflammation in a murine sepsis model via the ADAR1-miR-30a-SOCS3 axis. Mediat. Inflamm. 2020, 9607535. 10.1155/2020/9607535 32273831 PMC7128072

[B25] Singer M. Deutschman C. S. Seymour C. Shankar-Hari M. Annane D. Bauer M. (2016). The third international consensus definitions for sepsis and septic shock (Sepsis-3). JAMA 315, 801–810. 10.1001/JAMA.2016.0287 26903338 PMC4968574

[B26] Song J. Ren K. Zhang D. Lv X. Sun L. Deng Y. (2023). A novel signature combing cuproptosis- and ferroptosis-related genes in sepsis-induced cardiomyopathy. Front. Genet. 14, 1170737. 10.3389/FGENE.2023.1170737 37035738 PMC10076593

[B27] Sun K. Wu F. Zheng J. Wang H. Li H. Xie Z. (2024). Essential blood molecular signature for progression of sepsis-induced acute lung injury: integrated bioinformatic, single-cell RNA Seq and machine learning analysis. Int. J. Biol. Macromol. 282, 136961. 10.1016/j.ijbiomac.2024.136961 39481313

[B28] Toh T. S. Dondelinger F. Wang D. (2019). Looking beyond the hype: applied AI and machine learning in translational medicine. EBioMedicine 47, 607–615. 10.1016/J.EBIOM.2019.08.027 31466916 PMC6796516

[B29] Villar J. Ferrando C. Martínez D. Ambrós A. Muñoz T. Soler J. A. (2020). Dexamethasone treatment for the acute respiratory distress syndrome: a multicentre, randomised controlled trial. Lancet Respir. Med. 8, 267–276. 10.1016/S2213-2600(19)30417-5 32043986

[B30] Wang Y. M. Qi X. Gong F. C. Chen Y. Yang Z. T. Mao E. Q. (2020). Protective and predictive role of Mucin1 in sepsis-induced ALI/ARDS. Int. Immunopharmacol. 83, 106438. 10.1016/J.INTIMP.2020.106438 32247267

[B31] Wang X. Zhang C. Zou N. Chen Q. Wang C. Zhou X. (2022). Lipocalin-2 silencing suppresses inflammation and oxidative stress of acute respiratory distress syndrome by ferroptosis via inhibition of MAPK/ERK pathway in neonatal mice. Bioengineered 13, 508–520. 10.1080/21655979.2021.2009970 34969358 PMC8805876

[B32] Wang T. Kaneko S. Kriukov E. Alvarez D. Lam E. Wang Y. (2024). SOCS3 regulates pathological retinal angiogenesis through modulating SPP1 expression in microglia and macrophages. Mol. Ther. 32, 1425–1444. 10.1016/J.YMTHE.2024.03.025 38504518 PMC11081920

[B33] Wang B. Wang Y. Hu Z. Peng S. Li N. Dan Z. (2025). Enhancing Sirt1-mediated deacetylation of p62 with a self-assembling nanopeptide and resveratrol hydrogel to mitigate sepsis-induced inflammation. Phytomedicine 145, 157047. 10.1016/j.phymed.2025.157047 40627928

[B34] Weng D. Shi W. Hu Y. Su Y. Li A. Wei S. (2025). Neutralization of IL-33 ameliorates septic myocardial injury through anti-inflammatory, anti-oxidative, and anti-apoptotic by regulating the NF-κB/STAT3/SOCS3 signaling pathway. Biochem. Pharmacol. 237, 116954. 10.1016/j.bcp.2025.116954 40258576

[B35] Yang Y. Xie J. Guo F. Longhini F. Gao Z. Huang Y. (2016). Combination of C-reactive protein, procalcitonin and sepsis-related organ failure score for the diagnosis of sepsis in critical patients. Ann. Intensive Care 6, 51. 10.1186/S13613-016-0153-5 27287669 PMC4901212

[B36] Yasuda-Yamahara M. Rogg M. Yamahara K. Maier J. I. Huber T. B. Schell C. (2018). AIF1L regulates actomyosin contractility and filopodial extensions in human podocytes. PLoS One 13, e0200487. 10.1371/journal.pone.0200487 30001384 PMC6042786

[B37] Zhang Y. yu Ning B. tao (2021). Signaling pathways and intervention therapies in sepsis. Signal Transduct. Target Ther. 6, 407. 10.1038/S41392-021-00816-9 34824200 PMC8613465

[B38] Zhang W. Jiang H. Wu G. Huang P. Wang H. An H. (2023). The pathogenesis and potential therapeutic targets in sepsis. MedComm (Beijing) 4, e418. 10.1002/MCO2.418 38020710 PMC10661353

[B39] Zhang H. Meng F. Tang J. Zhang M. Jing Q. Peng G. (2025a). Lactate inhibits T-cell activation in sepsis through CD40LG downregulation and SOCS3-mediated JAK1/STAT3 pathway suppression. Biochim. Biophys. Acta Mol. Basis Dis. 1871, 167923. 10.1016/j.bbadis.2025.167923 40436284

[B40] Zhang W. Liu S. Ma W. Huang D. Li Y. (2025b). Mechanisms and clinical application prospects of curcumin in the treatment of sepsis. J. Inflamm. Res. 18, 9627–9636. 10.2147/JIR.S536551 40727460 PMC12301117

[B41] Zhao D. Liu Y. Xu Z. Shen H. Chen S. Zhang S. (2021). Integrative bioinformatics analysis revealed mitochondrial defects underlying hypoplastic left heart syndrome. Int. J. Gen. Med. 14, 9747–9760. 10.2147/IJGM.S345921 34934349 PMC8684406

[B42] Zheng Y. Wang J. Ling Z. Zhang J. Zeng Y. Wang K. (2023). A diagnostic model for sepsis-induced acute lung injury using a consensus machine learning approach and its therapeutic implications. J. Transl. Med. 21, 620. 10.1186/S12967-023-04499-4 37700323 PMC10498641

